# Modified Langenskiöld procedure for congenital patella dislocations in pediatric patients

**DOI:** 10.1186/s12891-022-05192-1

**Published:** 2022-03-12

**Authors:** Yueqiang Mo, Yanhui Jing, Dahui Wang, Dror Paley, Bo Ning

**Affiliations:** 1 Department of Orthopaedics Surgery, National Children’s Medical Center & Children’s Hospital of Fudan University, Wanyuan Road 399, Minhang District, Shanghai, 201102 China; 2 The Paley Advanced Limb Lengthening Institute at St. Mary’s Medical Center, 901 45th Street, Kimmel Building, West Palm Beach, FL 33407 USA

**Keywords:** Congenital patella dislocation, Modified Langenskiöld procedure

## Abstract

**Background:**

Great difficulty and more failures were the descriptions of the treatment of congenital patella dislocation in pediatric patients. This study aims to evaluate the outcomes of patients with congenital patellar dislocations treated with the modified Langenskiöld procedure.

**Methods:**

The medical records of 16 knees in 11 patients with a diagnosis of congenital patella dislocation were collected from September 2016 to March 2019. They were treated with the modified Langenskiöld procedure. The mean follow-up period was 37.8 months. The outcome measures were the Lysholm score, Kujala score, patellar stability, and knee range of motion.

**Results:**

Eleven patients, namely, eight girls and three boys, with 16 knees were enrolled. The mean age at the time of operation was 3.1 years. The post-operative mean Lysholm score was 94.8 (SD 5.1; 87–100), whereas the Kujala score was 95 (SD 5.9; 86–100). There were no recurrent dislocations, and all patients had full extension postoperatively.

**Conclusion:**

The modified Langenskiöld procedure is a promising solution for the treatment of congenital patella dislocations.

**Level of evidence:**

Level IV; Case Series; Treatment Study.

## Introduction

Congenital patella dislocation (CPD), a rare condition, is a pathological condition that manifests as permanent lateral dislocation of the patella. The true incidence of CPD is unknown since the confirmed cases are sporadic and the condition has many overlapping symptoms with other disorders such as Down syndrome, nail patella syndrome, and Meier-Gorlin syndrome. Moreover, CPD could be identified as a symptom of the aforementioned syndrome, adding more challenge to the diagnosis of the disorder [1].

Due to the pathological features of patients with CPD, including patellar dysplasia, trochlear dysplasia, vastus medialis obliquus pathology, surgery is the only effective treatment. Serial casting and bracing are effective in the treatment for flexion contracture of the affected knee, but the dislocated patella cannot be re-positioned without surgical intervention [2]. In addition, flexion contracture and genu valgum are typical findings that accompany CPD [2–4].

The Operation should be done as early as possible after diagnosis, helping make early correction which is important for avoiding sequelae. The age at which a patient undergoes surgical intervention depends on the age when the diagnosis is made [1, 2, 4]. Numerous surgical procedures have been developed to treat CPD. These procedures are usually subdivided into proximal, distal, and intra-articular procedures, which may be combined and tailored according to the underlying pathology. However, some techniques are complicated, requiring orthopedists to undergo longer training periods [5], whereas other techniques require extensive soft tissue separation [6, 7]. Nevertheless, the outcomes are not all satisfactory [7–9]. In 2015, Niedzielski reported a re-dislocation rate of 9%, poor to fair results accounting for 36% on the Lysholm score. In 2014, Camathias reported poor outcomes with a re-dislocation rate of 80% after the Stanisavljevic quadriceps transposition for the treatment of congenital and recurrent patellar dislocations [8].

In this report, we introduce the modified Langenskiöld procedure, which was first introduced by Paley to manage congenital femoral deficiency [10], and evaluate outcomes with a mean follow-up period of 37.8 months.

## Materials and methods

### Patients

Following approval from our Institutional Review Board, a retrospective study was conducted from September 2016 to March 2019. The ID of the patient diagnosed as congenital patellar dislocation was queried through the medical record system, and the detailed records and image data were searched according to the ID. The patients met the following inclusion criteria: (1) a diagnosis of congenital patella dislocation, (2) no history of knee surgery, and (3) received modified Langenskiöld procedure. The patients were excluded if their follow-up period was less than 2 year. Finally, 16 knees in 11 patients were included (Table 1). One senior pediatric orthopedist performed all the surgeries, and informed consent was obtained from all the patients’ guardians.

**Table 1:** Patient demographics and diagnoses

No	Sex	Age (year)	Laterality	Comorbidities	Concomitant procedures
1	M	6	L	/	EPCLR
2	F	4	R/L	bilateral knee flexion contractures; Spondyloepiphyseal dysplasia with congenital joint dislocation	EPCLR
3	F	1.5	L	Larsen syndrome; knee flexion contracture	EPCLR
4	F	3	L	knee flexion contracture	/
5	F	3	R	/	/
6	F	5	R/L	Dwarfism	EPCLR
7	F	1.5	L	Multiple joint fusion; Bilateral developmental dislocation of the Hip; right knee dislocation	EPCLR
8	M	3	R/L	Joint laxity	EPCLR
9	F	1.6	R/L	Down’s syndrome; Bilateral club feet	EPCLR
10	F	1.8	L	/	EPCLR
11	M	3.6	R/L	Joint laxity	EPLCR

*EPCLR* extra-articular posterior cruciate ligament reconstruction

### Surgical technique

With the patient in the supine position, the entire lower extremity was prepared, and a tourniquet was secured around the proximal thigh.

**Fig. 1:**
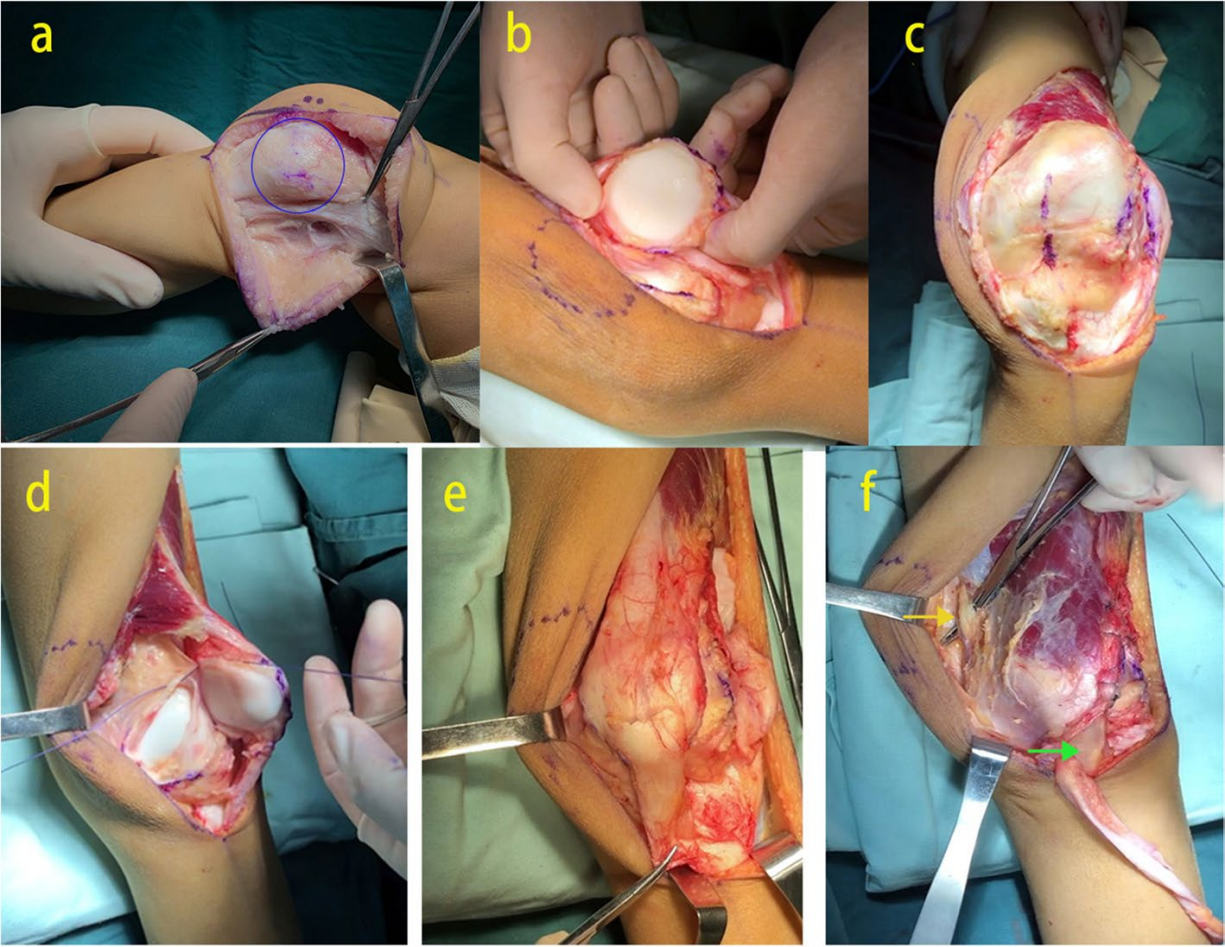
Modified Langenskiold & Grammont procedure. **a** The patella was dislocated laterally. **b** The patella was completely detached from the capsule and only attached to the quadriceps tendon and the patellar ligament. **c** A longitudinal line was generated on the synovium at the center of the new desired position for the patella. **d** An incision was created along the line, and the patella was circumferentially fixed to the synovium with a running absorbable suture. **e** The Q angle was significantly increased after the patella was medially relocated. We performed the Grammont procedure to decrease it. The patellar ligament was separated sharply from the tibial tuberosity, while keeping it attached to the periosteum distally. The insertion point was then shifted medially at least 1 cm and fixed to the medial periosteum by a suture. **f** The iliotibial band was harvested beforehand. After the completion of the modified Langenskiöld procedure, the iliotibial band was wrapped under the patellar ligament, passed through the intermuscular septum, folded around the adductor magnus tendon, and sutured back onto itself

The incision started approximately 5 cm proximal to the lateral condyle of the femur, extended along the lateral side of the dislocated patella, turned medial at the level of the tibial tubercle, and ended at the inferior pole of the tibial tubercle. A full thickness flap was created to expose the quadriceps femoris. The iliotibial band, the tendinous part of the vastus lateralis, and the posterior capsule were released. We observed the patella dislocated laterally (Fig. 1A). The capsule was incised and separated from the patella and synovium medially and laterally. The patellar ligament and quadriceps tendon were detached from the underlying synovium layer of the knee capsule without entering the knee, and the synovium layer was then circumferentially cut around the patella. The patella remained attached to only the quadriceps tendon and the patellar ligament (Fig. 1B).

After suturing the hole in the synovium layer in a longitudinal direction, a new longitudinal line was generated on the synovium at the center of the desired position for the patella (Fig. 1C). An incision was created at this new position, and the patella was secured circumferentially to the synovium with a running absorbable suture (Fig. 1D). The Q angle was significantly increased after the patella was medially relocated. Grammont patellar ligament realignment was performed to recover the Q angle. The patellar ligament was separated sharply from the tibial tuberosity, while keeping it attached to the periosteum distally. The insertion point was then shifted medially at least 1 cm and fixed to the medial periosteum (Fig. 1E). The medial capsule with the vastus medialis was advanced over the patella toward the lateral border and sutured, while the lateral capsule was left open. After releasing the tourniquet, the knee joint was moved to ensure that at least a 0° to 90° range of movement (ROM) remained and the patella was stable during this course. The wound was rinsed extensively with saline. Indwell negative pressure ball drainage was established, and the remaining deep and superficial layers were sutured.

### Concomitant procedures

For the patient with signs of genu valgum and joint instability, extra-articular posterior cruciate ligament reconstruction (EAPCLR) was performed. The iliotibial band was transected at the musculotendinous junction using the same incision created during the modified Langenskiöld procedure or an incision that was elongated by 3–4 cm proximally. To minimize the incision, we proceeded proximally by 4-5 cm with blunt dissection, when separated to the iliotibial band, which was stuck close to the surface of iliotibial band. Then the proximal end of the iliotibial band was cut by a scissor. It was reflected distally until its insertion to the tibia. After completing the modified Langenskiöld procedure, the iliotibial band was wrapped under the patellar ligament, passed through the intermuscular septum, folded around the adductor magnus tendon, and sutured back onto itself (Fig. 1F). If the knee flexion contracture was more than 15° before the operation and it was not significantly improved after releasing the lateral tissue and posterior capsule, then the peroneal nerve was decompressed at the neck of the fibula and the biceps tendon were released.

### Postoperative management

The knee was immobilized in a full-extension long leg splint. Two or 3 days later, the negative pressure ball was removed, and the splint was replaced with a tubular plaster cast. The first follow-up was 6 weeks after the operation. The cast was removed in the out-patient clinic, and progressive ROM was started. Subsequent follow-up was conducted at 1.5, 3, 6, 9, 18, and 24 months after surgery.

## Results

**Fig. 2:**
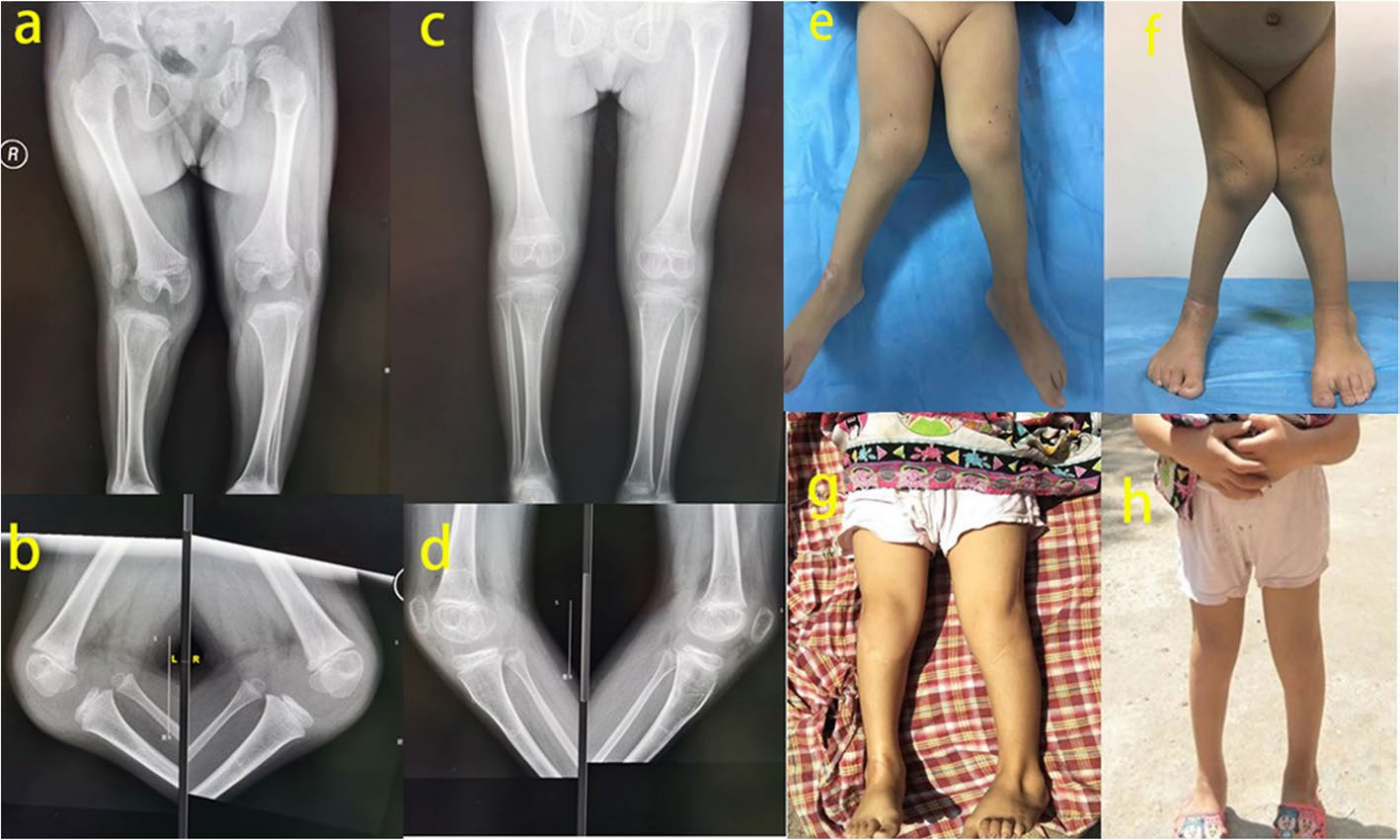
Preoperative and postoperative radiographic and Appearance manifestations. **a**, **b** X-rays before the operation indicated bilateral lateral dislocation of the patella and genu valgum of the right knee. **c**, **d** Eighteen months after the operation, genu valgum improved significantly on X-rays. **e**, **f**, **g**, **h** Appearance of the lower extremity pre- and postoperation

The mean age at the time of operation was 3.1 years. The mean follow-up time was 37.8 months (SD 2.5; 29–43). During this period, no patient experienced redislocation after the modified Langenskiöld procedure. Knee extension was limited by 15° to 25° in three patients (four knees) preoperatively. After the surgery, all the patients had normal extension. One patient had a limitation of flexion by 15° compared with the pre-operative status. The mean postoperative ROM was 0° to 135°. Genu valgum improved significantly after surgery (Fig. 2).

The mean postoperative Kujala score was 95 (SD 5.9; 86–100), and the Lysholm score was 94.8 (SD 5.1; 87–100). The children were asked if they experienced knee pain in their daily life, every time when they come for follow-up. Meanwhile, the opinions of their parents were also asked. No patient reported pain during activities of daily living. The results are summarized in Table 2.

**Table 2:** Clinical results of the patients

No	Re-dislocation	Kujala Score	Lysholm Score	Pain	ROM (degrees)
1	No	100	100	None	0–142
2	No	86/88	87/88	None	R:0–133, L:0–126
3	No	100	96	None	0–135
4	No	93	99	None	0–123
5	No	100	100	None	0–130
6	No	100/100	100/100	None	R:0–146, L:0–146
7	No	96	93	None	0–132
8	No	90/92	89/90	None	R:0–125, L:0–128
9	No	92/92	90/92	None	R:0–145, L:0–145
10	No	100	100	None	0–140
11	No	96/95	98/96	None	R:0–135, L:0–135

## Discussion

In Langenskiöld’s original description [2], the patella ligament on the proximal tibia is detached. Subsequently, the patella ligament is placed inside the synovium cavity and removed out of it from a hole distal to the first. The insertion is then buried in the hole in the tibial metaphysis and fixed by sutures. In this study, the outcomes were satisfactory, and no patient reported recurrent lateral dislocation after an average follow-up of > 3 years. Although no genu recurvatum was reported in the original study, in patients with open physes, surgery in the region of tibial tubercle can result in premature closure of the physis [11, 12]. In the modified Langenskiöld procedure we used, the tibial tubercle was intact, and the Q angle was decreased. In the present study, no growth disturbances occurred. Early weight bearing and early movement were possible. In contrast, bony distal re-alignment procedures bear the risk of non-union and require longer rehabilitation. In addition to this, the modified procedure left the patella and the tendon extra-synovial, which is similar to the physiological environment.

The “four in one” procedure includes lateral release, proximal “tube” realignment of the patella, semitendinosus tenodesis, and transfer of the patellar tendon [6, 7, 13]^.^ Semitendinosus tenodesis is performed with the semitendinosus tendon harvested and routed through an osseous tunnel in the patella. In our procedure, the patella was removed from the synovium and sutured in a medialized position of the synovium. It was stabilized in the synovial membrane, and the osseous tunnel in patella was not needed. Our procedure was much simpler and the patella was intact. The synovium was remarkably strong and inelastic, and the outcomes in our series were satisfactory.

Camathias reported a re-dislocation rate of 80% after the Stanisavljevic quadriceps transposition [8]. In the 11 patients (16 knees) in this study, no one reported recurrent dislocations and pain during the activities of daily life after an average follow-up of 3 years. The synovium is inelastic and strong enough to hold the patella. In our study, the mean postoperative Kujala score was 95 (SD 5.9; 86–100), and the Lysholm score was 94.8 (SD 5.1; 87–100). In 2012, Efe et.al. reported a 22% re-dislocation rate, the Kujala score was 85.0 (SD 14; 51–100) treated with Insall’s proximal patellar realignment procedure [14]. The increased Q angle is a risk factor for patellar instability [15–17]. To decrease the Q angle, which is significantly increased after the patella is medially relocated, we medialized the patellar ligament with the Grammont procedure, which is thought to be more suitable for skeletally immature patients. Genu valgum and knee joint hyperlaxity are also risk factors for patellar instability [16, 18, 19]. In this study, there were nine patients with knee joint hyperlaxity. They all received EPCLR, and a more stable joint was achieved after the procedure. In patients #2, #3, and #6, genu valgum was observed before the operation. Physical examinations and full-length lower extremity X rays indicated that joint hyperlaxity was the main cause of genu valgum in these patients, and distal femur deformity was the main cause in patient #6. Genu valgum improved significantly after EPCLR in these patients. In patient #6, it was not improved after EPCLR because of bone deformity caused by achondroplasia, so we performed hemi-epiphysiodesis of the medial distal part of the femur. At the latest follow-up, genu valgum was significantly improved.

Sever et al. [4] reported the outcomes of 12 patients (15 knees) with congenital or obligatory patellar dislocation treated with the Stanisavljevic quadriceps mechanism realignment procedure, which includes a proximal extensive subperiosteal realignment of the quadriceps mechanism, medial plication using the large overstretched medial capsule as a cover to the realigned patella, and an additional distal Roux-Goldthwait patellar tendon realignment procedure. Postoperative knee active extension was improved significantly for all patients, and no significant change in the flexion range was observed. Gao et al. [20]reported that all the patients had full range of movement without extensor lag but 12.2% of the patients experienced flexion limitations after surgery. We had three patients (four knees) with knee flexion contracture. They had a limitation of extension by 15° to 25°. Postoperative knee extension was improved significantly for three knees after releasing the posterior capsule, and one knee needed further peroneal nerve decompression, followed by release of the biceps tendon.

The flexion of the knee was limited to 123° in one patient, which was decreased by 15° postoperatively. The medialization of the patella and tendon lead to a tighter rectus femoris [16, 19]. If knee flexion was limited significantly during the operation, then the rectus femoris was released in case of flexion contracture after the operation. Two of the 16 knees in our research performed a release of the rectus femoris tendon. There were another two cases of superficial wound infection due to incision dehiscence. They were resolved after treatment with oral antibiotics and dressing changes. An extensive separation can disrupt the blood supply of the flap, and this might have been one reason for incision dehiscence.

The present study had some limitations. The follow-up of 37.8 months was relatively short compared with similar studies [2, 8, 11, 21, 22]. The small number of patients was another limitation. We will continue the follow-up and enroll more patients in the future.

## Conclusion

Treatment of congenital patella dislocation is complicated. The surgical procedure should be tailored to the specific pathological features of each patient. The modified Langenskiöld procedure provided a promising correction for CPD.

## Data Availability

The datasets generated and/or analysed during the current study are not publicly available due to limitations of ethical approval involving the patient data and anonymity but are available from the corresponding author on reasonable request.

## Abbreviations

CPD: Congenital patella dislocation
ROM: Range of movement
EAPCLR: Extra-articular posterior cruciate ligament reconstruction
